# The safety and efficacy of TACE combined with HAIC, PD-1 inhibitors, and tyrosine kinase inhibitors for unresectable hepatocellular carcinoma: a retrospective study

**DOI:** 10.3389/fonc.2024.1298122

**Published:** 2024-01-22

**Authors:** Zhongjing Huang, Ziyi Wu, Lidong Zhang, Likun Yan, Hai Jiang, Junhua Ai

**Affiliations:** Department of General Surgery, The First Affiliated Hospital, Jiangxi Medical College, Nanchang University, Nanchang, Jiangxi, China

**Keywords:** hepatocellular carcinoma, transarterial chemoembolization, immunotherapy, hepatic artery infusion chemotherapy, targeted therapy

## Abstract

**Objective:**

To assess the effectiveness and safety of transarterial chemoembolization (TACE) in combination with hepatic artery infusion chemotherapy (HAIC)、PD-1 inhibitors, and tyrosine kinase inhibitors(TKI) for unresectable hepatocellular carcinoma (HCC).

**Methods:**

A retrospective analysis was performed on 158 unresectable HCC patients admitted to the First Affiliated Hospital of Nanchang University between May 2019 and October 2022. The patients were split into two groups based on the type of treatment they received: TACE combined with HAIC,PD-1 and TKI group (THPK) and TACE combined with PD-1 and TKI group (TPK). The response was evaluated using modified solid tumor Efficacy Assessment Criteria (mRECIST). Kaplan-Meier curves were used to analyze the overall survival (OS). OS-influencing factors were identified using the Cox proportional risk regression model.

**Results:**

Finally, 63 patients who received THPK treatment and 60 patients who had TPK treatment were included. The THPK group had higher DCR (77.78% vs. 55.00%, P=0.007) and ORR (20.63% vs. 13.34%, P=0.282) than the TPK group did. The survival analysis curve also showed that the median OS was substantially longer in the THPK group than in the TPK group (OS: 21 months vs. 14 months, P=0.039). After multivariate Cox regression-corrected analysis, extrahepatic metastases (P=0.002) and methemoglobin >400 (P=0.041) were adverse influences on OS, but the THPK group (relative to the TPK group) was an independent favorable prognostic factor for OS (P=0.027). The results of the subgroup analysis showed that the addition of HAIC therapy to TPK treatment in patients with BCLC stage C, age ≦60 years, ECOG grade 0 and lobular distribution of tumors prolonged overall survival time and improved prognosis. Except for nausea, there was no difference in the adverse events between the two groups.

**Conclusion:**

In patients with unresectable HCC, the THPK group had a longer OS and similar adverse events compared to the TPK group. In the future, TACE-HAIC in combination with targeted and immunotherapy may be a more effective therapeutic option for hepatocellular carcinoma that cannot be surgically removed.

## Introduction

Hepatocellular carcinoma (HCC) is the most common primary malignancy affecting the liver and is the fourth most common cause of cancer-related deaths worldwide (1). Around 50% of patients with advanced hepatocellular carcinoma do not receive surgery, despite advances in medical care (2). The first-line therapy for advanced hepatocellular carcinoma is now tyrosine kinase inhibitors like lenvatinib and sorafenib, which are efficient and well-tolerated in a randomized III controlled clinical trial (3). Moreover, clinical trials have demonstrated that transcatheter interventional chemoembolization in combination with tyrosine kinase inhibitors(TKI)and programmed cell death protein-1 (PD-1) improves overall survival and disease control rates in advanced HCC (4). Hepatic artery infusion chemotherapy has drawn attention recently due to its high remission rates and good survival rates in advanced HCC (5), Numerous randomized clinical trials have demonstrated that HAIC therapy has superior survival outcomes to sorafenib monotherapy (6, 7). Little research has been done so far on the effectiveness of THPK therapy. To establish a standard of care for the treatment of advanced HCC, we created this retrospective study to assess the safety and effectiveness of THPK and TPK in treating patients with advanced HCC.

## Methods

### Patient characteristics

**Figure 1:**
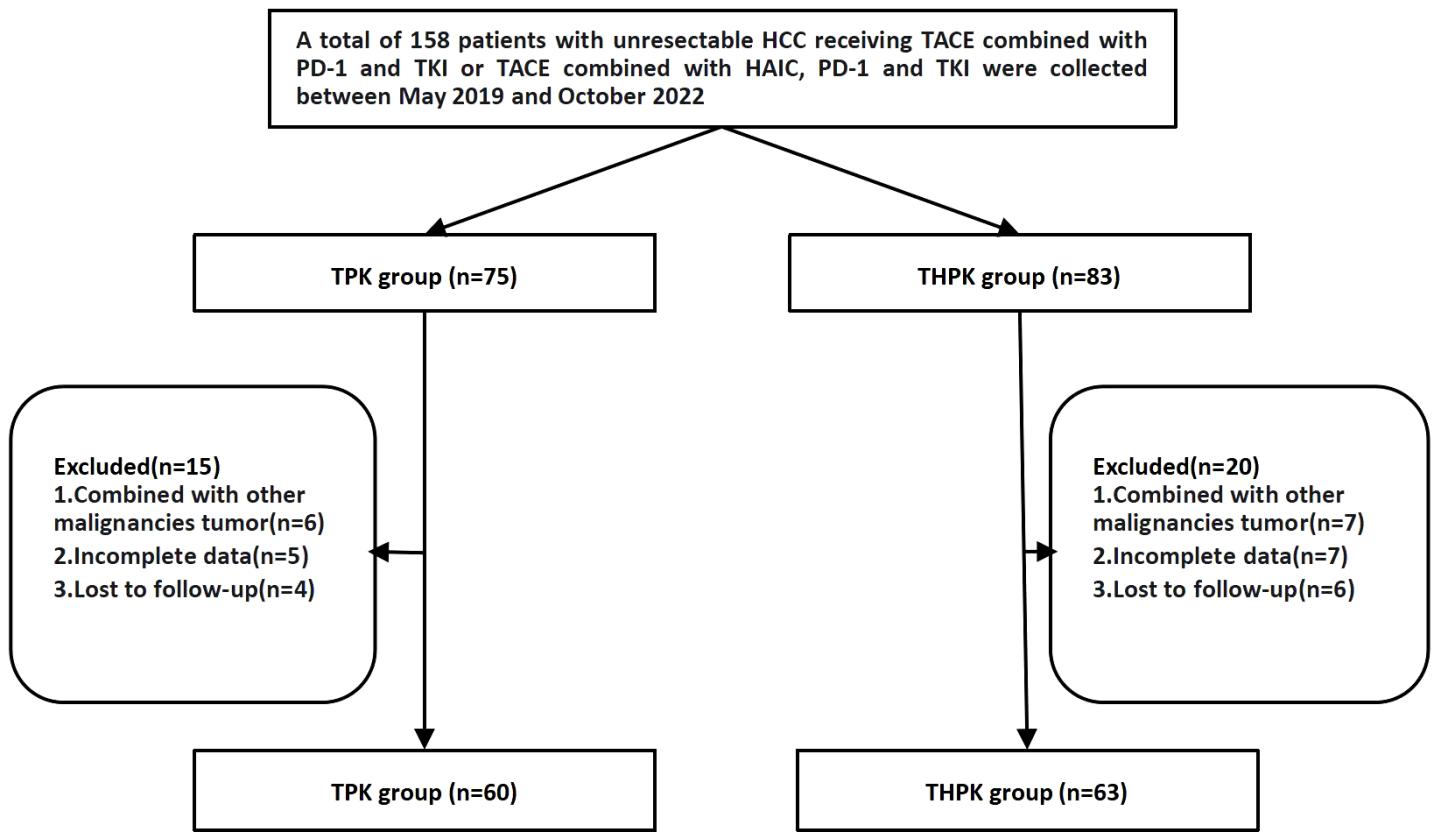
Flow diagram summarizing the disposition of patients.

Between May 2019 and October 2022, 158 patients with unresectable HCC who underwent first TPK and THPK treatment (In clinical care unresectable hepatocellular carcinoma was randomized to these two different treatment modalities) at the First Affiliated Hospital of Nanchang University were identified. A total of 123 patients were finally included according to the following inclusion criteria: (1) clinically and pathologically confirmed HCC; (2) progressive disease ineligible for radical surgery; (3) patients over the age of 18; (4) cirrhotic Child-Pugh grades A and B; (5) Eastern Cooperative Oncology Group (ECOG) PS score of 0-2; (6) complete follow-up data; and (7) informed consent form signers. The following exclusion criteria were used to weed out patients: (1) those who were contraindicated for TACE and HAIC; and (2) those who had a combined history of other malignancies (**Figure 1**).

### Data collection

The First Affiliated Hospital of Nanchang University’s archival medical records were the source of all clinical information needed for diagnosis. Gender, age, cirrhosis, liver function grade, maximum tumor diameter, ECOG grade, venous cancer thrombosis, extrahepatic metastases, preoperative total bilirubin (TBIL), alpha-fetoprotein (AFP), Barcelona (BCLC) stage, and the number of TACE and HAIC treatments were among the variables that were gathered and examined. The last check-in took place on June 1, 2023.

OS was outlined as the period of time from the start of the first TACE or TACE in conjunction with HAIC treatment until the time of death or the last follow-up. According to the Response Evaluation Criteria in Solid Tumors (RECIST) (8), two hepatobiliary surgeons assessed the tumor response. All objective tumor remissions were verified at least four weeks after the initial treatment.

### Transcatheter hepatic artery embolization chemotherapy

Cannulation along the femoral artery to the hepatic artery or its branches was accomplished using the Seldinger technique. After angiography confirms the tumor’s location, number, size, and blood supply, the tumor donor artery is super-selected for annulations. First, chemotherapeutic agents were infused through the tumor donor artery by putting 30 mg of epirubicin hydrochloride or idarubicin hydrochloride and oxaliplatin (60 mg/m^2^) or raltitrexed (4 mg) during TACE; second, the tumor was embolized with iodinated oil mixed with chemotherapeutic agents in amounts ranging from 3 to 20 ml of iodinated oil. Third, 8-sphere (300-500 um) blank microspheres were added to embolize all remaining tumor supplying arteriesembolization (9). During the treatment, the treatment was tailored to the tumor’s location, size, and number.

### Hepatic artery perfusion chemotherapy

Selective placement of the microcatheter into the tumor-supplying artery. If necessary, the gastroduodenal artery is occluded. The microcatheter is then connected to an arterial perfusion pump to administer the following treatment (FOLFOX): first continuous arterial line infusion of 85 mg/m2 oxaliplatin for 2 hours, followed by constant arterial line infusion of 300 mg/m2 calcium Levofolinate for 2 hours after the infusion is completed, and finally continuous arterial line infusion of 2400 mg/m2 fluorouracil for 46 hours (10). If poorly tolerated during the HAIC course, treatment was discontinued.

### PD-1 inhibitors and tyrosine kinase inhibitors

Regarding drug accessibility, the TKIs in this study are sorafenib, lenvatinib, regorafenib, apatinib, and donafenib, and the PD-1 inhibitors are certolizumab, ocrelizumab, sintilimab, and mepolizumab. The doses of these drugs are administered according to guidelines and will be adjusted based on physical condition, liver function, and treatment tolerance.

### Data analysis

The chi-square test was used to compare all variables that were categorical. The Kaplan-Meier method was used to analyze the OS curves, and the log-rank test results were used to compare group differences. A Cox regression model was used for multivariate analysis for variables significant in the univariate analysis, and P values of 0.05 were considered significant. SPSS version 26.0 was used for all statistical analyses.

## Results

### Patient characteristics

Clinical and imaging data are shown in **Table 1**. 123 patients with unresectable HCC were eventually included in the study, 60 of whom received TPK and 63 received THPK treatment. The cut-off value was the final follow-up time on June 1, 2023.

**Table 1:** Characteristics of patients.

Characteristic	TPK group	THPK group	χ^2^	P value
Gender				
male	52	57	0.442	0.506
female	8	6		
Age(year)				
≦60	34	43	1.762	0.184
>60	26	20		
Hbv-infection				
yes	50	46	1.909	0.167
no	10	17		
Liver cirrhosis				
yes	48	48	0.260	0.610
no	12	15		
Child-Pugh score				
A	41	39	0.559	0.455
B	19	24		
Tumor number				
Solitary	18	14	0.996	0.326
multiple	42	49		
Tumor distribution				
uni-lobar	18	14	1.430	0.232
bi-lobar	42	49		
Maximum tumor diameter(cm)				
≦10	35	34	0.238	0.626
>10	25	29		
ECOG				
0	31	28	0.642	0.423
1	29	35		
Venous cancer embolism				
yes	22	24	0.027	0.870
no	38	39		
Extrahepatic metastases				
yes	21	24	0.127	0.722
no	39	39		
BCLC stage				
B stage	31	26	1.336	0.248
C stage	29	37		
Preoperative total bilirubin				
≦26 umol/L	47	48	0.080	0.777
>26 umol/L	13	15		
Preoperative albumin				
>30 g/L	47	50	0.020	0.889
≦30 g/L	13	13		
AFP				
≦400 ng/mL	33	39	0.604	0.437
>400 ng/mL	27	24		
Number of treatments				
≦2	33	28	1.370	0.242
>2	27	35		

AFP, alpha fetoprotein. TPK, TACE combined with PD-1 and TKI group; THPK, TACE combined with HAIC,PD-1 and TKI group.

### OS, ORR, DCR, subgroup analysis and tumor response in all patients

**Figure 2:**
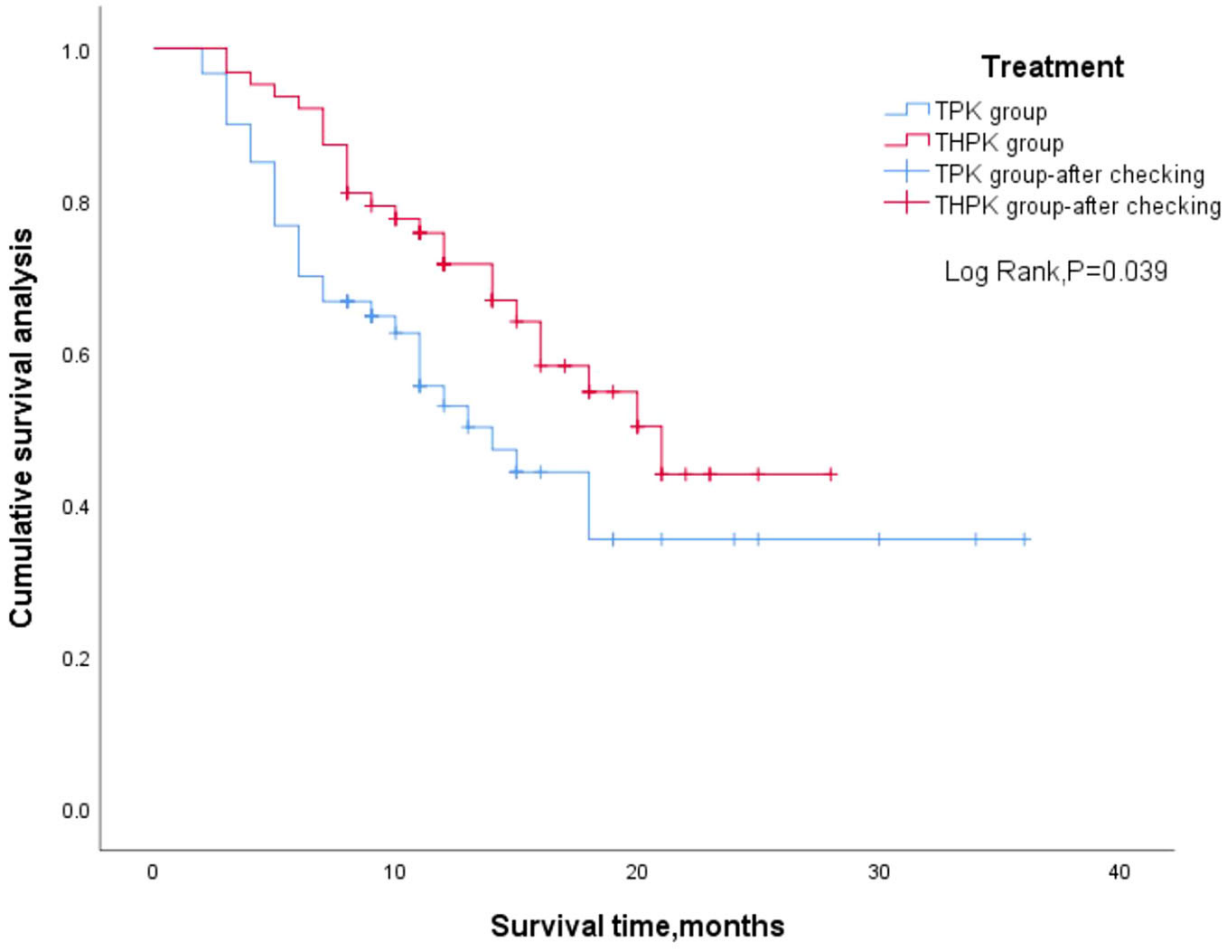
Two groups of patients’ overall survival outcome of Kaplan Meier curve. TPK, TACE combined with PD-1 and TKI group; THPK, TACE combined with HAIC, PD-1 and TKI group.

**Figure 3:**
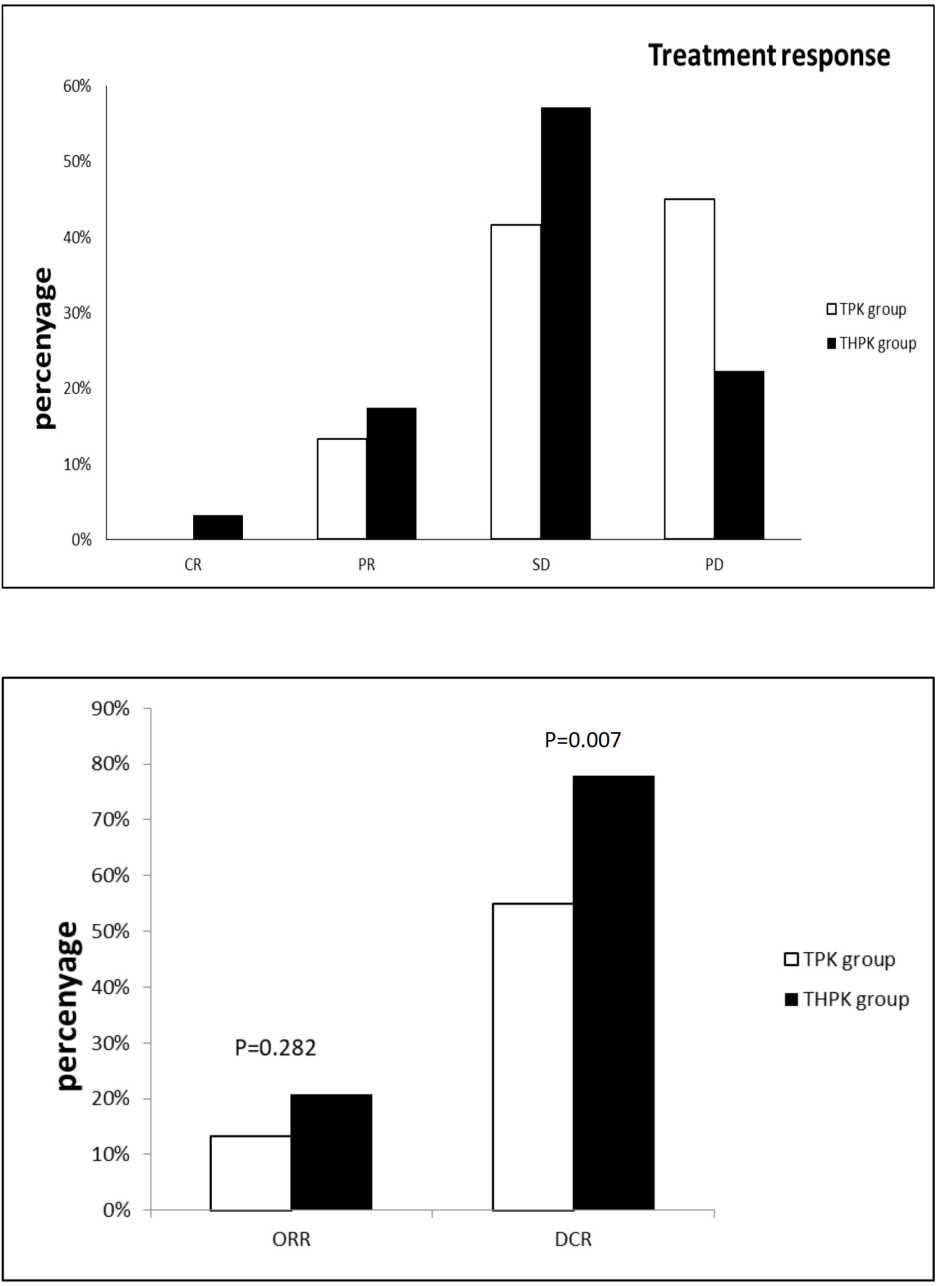
Histograms of unresectable HCC remission,ORR and DCR using RECIST criteria in the THPK and TPK groups. TPK group,TACE combined with PD-1 and TKI;THPK group,TACE combined with HAIC,PD-1 and TKI;CR, complete response;PR, partial response; SD, stable disease;PD, progressive disease; ORR, objective response rate; DCR, disease control rate.

For the total study cohort, the median follow-up lasted 11 months (range: 2–36 months). 25 patients (39.7%) in the THPK group and 31 patients (51.7%; P = 0.182) passed away during the follow-up period. The median OS in the THPK group was 21 months (range: 14.623 months-27.377 months), while in the TPK group it was 14 months (range: 8.940 months-19.060 months), according to survival curve analysis (**Figure 2**). According to the mRECIST criteria, the ORR was 20.63% and 13.34% (P = 0.282) and the DCR was 77.78% and 55.00% (P = 0.007) in the THPK and TPK groups, respectively, as shown in **Table 2** and **Figure 3**.

**Table 2:** Treatment response.

Response	Patients, No. (%)		P value
	TPK group(N=60)	THPK group(N=63)	
CR	0(0.00)	2(3.17)	
PR	8(13.34)	11(17.46)	
SD	25(41.66)	36(57.14)	
PD	27(45.00)	14(22.23)	
ORR	8(13.34)	13(20.63)	0.282
DCR	33(55.00)	49(77.78)	0.007

CR, complete response; PR, partial response; SD, stable disease; PD, progression disease; ORR, objective response rate; DCR, disease control rate; ORR = CR + PR; DCR = CR + PR + SD.

All clinical factors were prognostically analyzed using Cox regression analysis (**Table 3**). Following multifactorial analysis, extrahepatic metastases and methemoglobin levels above 400 were found to be unfavorable prognostic factors for OS, while treatment (THPK vs. TPK, HR= 0.541; 95% confidence interval[CI]0.315-0.931; P = 0.027) was an independent favorable prognostic factor for OS.

**Table 3:** Univariate and multivariate Cox’s regression analysis for OS.

Characteristic	Overall survival					
	Univariate analysis			Multivariate analysis		
	HR	95%CI	P value	HR	95%CI	P value
Treatment (THPK vs TPK)	0.582	0.343-0.988	0.045	0.541	0.315-0.931	0.027
Gender (male vs female)	0.549	0.259-1.163	0.117	0.506	0.232-1.104	0.087
Age (>60vs≦60)	0.711	0.402-1.257	0.241			
Hbv-infection (yes vs no)	1.532	0.771-3.046	0.224			
Liver cirrhosis(yes vs no)	1.313	0.678-2.544	0.419			
Child-Pugh score(A vs B)	0.835	0.485-1.436	0.514			
Tumor number (multiple vs Solitary)	0.754	0.426-1.334	0.332			
Tumor distribution(bi-lobar vs uni-lobar)	1.234	0.651-2.336	0.519			
Maximum tumor diameter(>10 vs≦10)	1.048	0.619-1.773	0.862			
ECOG staging (1 vs 0)	1.501	0.880-2.558	0.136	1.151	0.635-2.084	0.643
Venous cancer embolism (yes vs no)	1.578	0.931-2.675	0.090	1.544	0.738-3.230	0.249
Extrahepatic metastases(yes vs no)	3.006	1.756-5.146	<0.001	3.489	1.582-7.695	0.002
BCLC stage(C stage vs B stage)	2.055	1.180-3.580	0.011	0.714	0.246-2.072	0.535
Preoperative total bilirubin(>26 vs≦26)	1.018	0.546-1.899	0.955			
Preoperative albumin (>30 vs≦30)	0.719	0.393-1.317	0.286			
AFP (>400 vs≦400)	1.825	1.077-3.092	0.025	1.749	1.022-2.991	0.041
Number of treatments (>2 vs≦2)	0.784	0.461-1.332	0.367			

AFP, α-­fetoprotein; CI, confidence interval; ECOG, Eastern Cooperative Oncology Group; BCLC, Barcelona Clinic Liver Cancer; HBV, hepatitis B virus; HR, hazard ratio.

Subgroup analysis was performed after grouping all clinical factors (**Table 4**), and the results showed that for BCLC stage C (HR = 0.436, 95% CI (0.227-0.837), P = 0.013), age≦60 years (HR = 0.442, 95% CI (0.234-0.8360), P = 0.012), ECOG grade 0 (HR= 0.351,95%CI (0.145-0.851), P = 0.021), and patients with lobulated tumors (HR=0.525,95%CI (0.289-0.9530, P = 0.034), the addition of HAIC treatment to TPK treatment prolonged overall survival time and improved prognosis.

**Table 4:** Subgroup analysis of clinicopathological factors affecting the overall survival of patients.

Characteristic	THPK group	TPK group	HR	95%CI	P value
Gender					
male	57	52	0.655	0.371-1.155	0.144
female	6	8	0.340	0.068-1.703	0.189
Age(year)					
≦60	43	34	0.442	0.234-0.836	0.012
>60	20	26	0.921	0.354-2.396	0.866
Hbv-infection					
yes	46	50	0.646	0.359-1.160	0.144
no	17	10	0.452	0.131-1.568	0.211
Liver cirrhosis					
yes	48	48	0.579	0.320-1.048	0.071
no	15	12	0.658	0.198-2.192	0.496
Child-Pugh score					
A	39	41	0.600	0.306-1.176	0.137
B	24	19	0.550	0.233-1.299	0.173
Tumor number					
Solitary	14	18	0.380	0.133-1.086	0.071
multiple	49	42	0.707	0.377-1.328	0.281
Tumor distribution					
uni-lobar	13	18	0.745	0.236-2.358	0.617
bi-lobar	50	42	0.525	0.289-0.953	0.034
Maximum tumor diameter(cm)					
≦10	34	35	0.550	0.265-1.144	0.110
>10	29	25	0.629	0.290-1.362	0.239
ECOG					
0	28	31	0.351	0.145-0.851	0.021
1	35	29	0.756	0.381-1.501	0.424
Venous cancer embolism					
yes	24	22	0.469	0.210-1.046	0.064
no	39	38	0.660	0.325-1.342	0.251
Extrahepatic metastases					
yes	24	21	0.503	0.243-1.040	0.064
no	39	39	0.605	0.278-1.317	0.206
BCLC stage					
B stage	26	31	0.698	0.279-1.748	0.443
C stage	37	29	0.436	0.227-0.837	0.013
Preoperative total bilirubin					
≦26 umol/L	48	47	0.617	0.338-1.128	0.117
>26 umol/L	15	13	0.492	0.163-1.490	0.210
Preoperative albumin					
>30 g/L	50	47	0.681	0.371-1.250	0.215
≦30 g/L	13	13	0.358	0.119-1.077	0.068
AFP					
≦400 ng/mL	39	33	0.663	0.311-1.413	0.288
>400 ng/mL	24	27	0.515	0.243-1.095	0.085
Number of treatments					
≦2	28	33	0.495	0.224-1.090	0.081
>2	35	27	0.683	0.329-1.418	0.306

AFP, α-­fetoprotein; CI, confidence interval; ECOG, Eastern Cooperative Oncology Group; BCLC, Barcelona Clinic Liver Cancer; HBV, hepatitis B virus; HR, hazard ratio.

### Comparison of complications after treatment

Complications were assessed in both patient groups. None of the participants who were enrolled in the study experienced any fatal complications. The most frequent side effects included nausea, vomiting, malaise, brief fever, and brief elevation in blood pressure, hepatic dysfunction, and bone marrow suppression. Less frequently occurring side effects included hypothyroidism, rash, dysuria, and hypothyroidism, all of which could be managed with symptomatic therapy. In comparison to the TPK group, nausea occurred more frequently in the THPK group (P = 0.005, **Table 5**).

**Table 5:** Adverse events in the TPK and THPK groups.

Adverse reaction	TPK group (N=60)	THPK group (N=63)	P value
Abdominal pain	46	46	0.641
Nausea	8	22	0.005
vomiting	1	2	1.000
Fatigue	15	8	0.080
Hypothyroidism	3	2	0.956
Rash	0	1	1.000
Difficulty in urination	5	12	0.085
Hypertension	17	11	0.151
Fever	18	24	0.344
Decreased platelets	33	34	0.909
Decreased white blood cells	12	20	0.138
Increased total bilirubin	18	24	0.344
ALT increased	38	47	0.176
AST increased	46	52	0.418

ALT, alanine aminotransferase; AST, aspartate aminotransferase.

## Discussion

With greater median OS, ORR, and DCR in the THPH group compared to the TPK group, our trial demonstrated a more substantial survival benefit in unresectable hepatocellular carcinoma in the THPK group compared to the TPK group. According to a multifactorial analysis, extrahepatic metastases and methemoglobin levels greater than 400 had a negative impact on OS while therapy in the THPK group was an independent prognostic factor that was positive. These indicate that the THPK group may be a more effective treatment option for hepatocellular carcinoma that is unresectable.

For advanced HCC, several trials have examined the effectiveness and safety of local treatment (TACE or HAIC) combined with targeted and immunotherapy (11, 12).However, the local treatment modality in previous studies was mainly TACE or HAIC alone, while this study focuses on the local treatment modality of TACE combined with HAIC. One study found that TACE combined with HAIC demonstrated higher surgical conversion rates and PFS and OS benefits than TACE alone in patients with unresectable HCC (13, 14). Our study validated the efficacy of TACE combined with HAIC combined with targeted therapy and immunotherapy in patients with unresectable hepatocellular carcinoma. In China, TACE continues to be the mainstay of local treatment for hepatocellular carcinoma that is unrespectable. In addition, there are cases that some patients with unrespectable hepatocellular carcinoma can obtain an increase in liver volume after double TACE treatment failure followed by associated liver partition and portal vein ligation for staged hepat-ectomy (ALPPS) (15). However, a meta-analysis showed that the FOLFOX-HAIC regimen for unresectable hepatocellular carcinoma may be more effective than TACE (16, 17). HAIC is a continuous infusion of chemotherapy through the tumor supply artery, which significantly increases the local drug concentration and exerts anti-tumor effects in the liver and hepatic tumors. Additionally, PD-1 inhibitors and tyrosine kinase inhibitors have been found in several clinical studies to have effective anti-tumor effects when used in the treatment of advanced HCC (18, 19). In addition, in second-line treatment of some patients with refractory advanced HCC, regorafenib and cabozantinib were associated with longer OS compared to placebo, while in patients with AFP >400 ng/mL, regorafenib, cabozantinib and remolizumab were associated with longer PFS and OS compared to placebo (20, 21).Therefore, TACE combined with HAIC, targeted therapy, and immunotherapy may serve as a new treatment modality for patients with unresectable HCC.

The effect of the combination treatment used in this study may be related to several factors:1)TACE results in tissue hypoxia and local medication delivery of cytotoxic agents, which produces an increase in vascular endothelial growth factor (VEGF), which may trigger tumor revascularization and local recurrence (22). The tumor microenvironment is immunosuppressed by PD-1 inhibitors so that it can be reprogrammed to react to immune checkpoint inhibition and restore normalcy (23).2)TKI can block VEGFR, which prevents tumor neovascularization, and further normalize the tumor microenvironment, which was previously blocked by VEGF, triggering an efficient immune response against the tumor (23, 24).3)HAIC therapy can directly administer high doses of anti-cancer medications to highly vascular HCC, including micro-metastases that are difficult to identify with imaging and may not have a significant arterial blood supply (25), Additionally, it appears to prevent tumor metastasis and growth. In patients with unresectable HCC, the combination of TACE, HAIC, TKI, and PD-1 inhibitors may therefore result in synergistic anticancer activity and aid to enhance clinical outcomes. It is essential to highlight that earlier studies (4) assessed a median OS of 16.9 (95% confidence interval [CI] 14.9-18.8) months in patients with unresectable HCC treated with a combination of TACE, TKI, and PD-1 inhibitors. This appears to be a longer OS than the control patients treated with TACE,TKI,and PD-1 inhibitors in our study, which may be related to the higher number of stage C in the BCLC staging in this study. In our study, the THPK group had a greater ORR and DCR than the TPK group throughout therapy remission. The THPK group’s efficacy in treating unresectable hepatocellular carcinoma was demonstrated by the multifactorial Cox analysis, which revealed that the THPK group was an independent prognostic indicator of OS.

In our study, we found that for BCLC stage C (HR=0.436,95%CI(0.227-0.837),P=0.013), age≦60 years (HR= 0.442, 95% CI (0.234-0.8360), P = 0.012), ECOG grade 0 (HR =0.351,95% CI (0.145-0.851), P = 0.021), and patients with lobulated tumors (HR = 0.525, 95% CI (0.289-0.9530, P = 0.034) who underwent THPK had better overall survival time. In the future, personalized therapeutic regimens could be provided for the above specific unresectable hepatocellular carcinoma populations.

All adverse events were controllable in our trial and were comparable to previously published data for each treatment (4, 26, 27) for the THPK group against the TPK group. There were no brand-new or unexpected negative events noted. Except for nausea, both groups had adverse events throughout treatment at comparable rates and intensities. These findings imply that the safety profile of the THPK group was acceptable because both the THPK group and the TPK group were tolerated, and the THPK group did not have a significantly higher risk of adverse events than the TPK group. This study does have some drawbacks. As a single-center retrospective analysis, there was unavoidable selection bias in this study. Second, the large range of TKI and PD-1 inhibitors has an impact on the uniformity of treatment methods. Third, this study’s follow-up period was quite brief. The results of this investigation should be broadened to a multicenter study in order to achieve a higher degree of medical evidence because the number of cases was relatively low. In conclusion, our study showed that the THPK group was safe and effective at treating patients with unresectable HCC. In comparison to the TPK group, these patients experience much greater therapeutic response and longer survival thanks to THPK group therapy. A prospective, randomized controlled experiment with a sizable sample size is required to confirm these findings.

## Conclusion

TACE combined with HAIC, PD-1 and TKI therapy may be a better treatment modality for unresectable HCC. Of course, more clinical trials are needed to verify this in the future.

## Data availability statement

The raw data supporting the conclusions of this paper will be Provided by the authors without reservation. Detailed see https://www.jianguoyun.com/p/DSKLfnYQp8GmDBiqm7IFIAA.

## Ethics statement

As this is an observational study, the Research Ethics Committee of the First Affiliated Hospital of Nanchang University has confirmed that ethical approval is not required.

## Author contributions

ZH: Data curation, Formal analysis, Writing – original draft, Writing – review & editing. ZW: Data curation, Formal analysis, Writing – original draft. LZ: Data curation, Formal analysis, Writing – original draft. LY: Data curation, Formal analysis, Writing – original draft. HJ: Writing – original draft, Writing – review & editing. JA: Writing – original draft, Writing – review & editing.
